# Economic Burden of Acute Gastroenteritis among Members of Integrated Healthcare Delivery System, United States, 2014–2016

**DOI:** 10.3201/eid3005.230356

**Published:** 2024-05

**Authors:** John F. Dickerson, Suzanne B. Salas, Judy Donald, Holly C. Groom, Mi H. Lee, Claire P. Mattison, Aron J. Hall, Mark A. Schmidt

**Affiliations:** Center for Health Research, Kaiser Permanente Northwest, Portland, Oregon, USA (J.F. Dickerson, S.B. Salas, J. Donald, H.C. Groom, M.H. Lee, M.A. Schmidt);; Cherokee Nation Assurance, Arlington, Virginia, USA (C.P. Mattison);; Centers for Disease Control and Prevention, Atlanta, Georgia, USA (C.P. Mattison, A.J. Hall)

**Keywords:** acute gastroenteritis, enteric infections, viruses, bacteria, economic, healthcare delivery system, MAAGE, cost, surveillance study, United States

## Abstract

We conducted a large surveillance study among members of an integrated healthcare delivery system in Pacific Northwest of the United States to estimate medical costs attributable to medically attended acute gastroenteritis (MAAGE) on the day care was sought and during 30-day follow-up. We used multivariable regression to compare costs of MAAGE and non-MAAGE cases matched on age, gender, and index time. Differences accounted for confounders, including race, ethnicity, and history of chronic underlying conditions. Analyses included 73,140 MAAGE episodes from adults and 18,617 from children who were Kaiser Permanente Northwest members during 2014–2016. Total costs were higher for MAAGE cases relative to non-MAAGE comparators as were costs on the day care was sought and costs during follow-up. Costs of MAAGE are substantial relative to the cost of usual-care medical services, and much of the burden accrues during short-term follow-up.

Medically attended acute gastroenteritis (MAAGE) is a substantial driver of health services use by patients, accounting for >10 million outpatient encounters and 1 million hospitalizations each year (*1*,*2*). Initial MAAGE encounters in primary care and urgent need departments impart an immediate burden on healthcare systems (*3*). In addition, MAAGE-related hospitalizations may persist for multiple days, and patients may return for additional MAAGE services if symptoms worsen or fail to abate. Determining the financial impact of MAAGE on health systems and their members would provide important information about the potential value of interventions targeting acute gastroenteritis (AGE).

Several studies in Europe and the United States have evaluated the costs of AGE and of MAAGE, with a focus on the costs attributable to the onset and duration of an MAAGE episode. Researchers in Belgium estimated that MAAGE episodes accounted for direct costs of €112 million ($126 million in 2017 US dollars) in Belgium annually (*4*), and researchers in Switzerland estimated that healthcare costs resulting from AGE in combination with campylobacteriosis amounted to €29–45 million ($33–51 million in 2017 US dollars) in Switzerland annually (*5*). A study estimating the impact of MAAGE among a managed care population in the United States estimated a substantial annual burden of MAAGE episodes to health systems of $3.88 billion and demonstrated that the costs attributed to AGE increased ≈26% during 2006–2011 (*6*). However, those prior studies did not include subsequent, short-term costs attributable to AGE through exacerbation of underlying conditions or illnesses or services for lingering symptoms or sequelae. As has been recently shown (*7*), much of the burden associated with an AGE episode could be experienced during the short-term follow-up period. Given the high prevalence of MAAGE encounters and the acute need for medical resources to treat AGE episodes, additional data on short-term costs would help to more comprehensively quantify the economic costs of MAAGE encounters and assess potential economic benefits of prevention of or early intervention in AGE episodes.

We evaluated the economic burden of MAAGE on a fully integrated healthcare delivery system serving members residing in the Pacific Northwest of the United States. As part of a large surveillance study of AGE, we quantified initial medical expenditures at the time care was sought, as well as all ongoing short-term medical costs during a 30-day follow-up period (*8*). We used data from electronic health records (EHR) of health system members identified with MAAGE and of matched members without MAAGE who had a health system visit (either outpatient, emergency, or telephone encounters) during the same time period, with the aim of estimating both the same-day and incremental 30-day health care expenditures associated with MAAGE. We evaluated health care expenditures separately for children and adults and further stratified analyses by age among both children (0–4 years and 5–17 years) and adults (18–64 years and ≥65 years).

## Methods

### Data Source

We used EHR data from Kaiser Permanente Northwest (KPNW), a fully integrated health care delivery system serving ≈616,000 medical members located primarily in Oregon and southwest Washington. Those data, stored in the research data warehouse at the KPNW Center for Health Research in Oregon contained information on diagnoses, physical findings, tests, procedures, medications, insurance enrollment, claims, state mortality records, and census-derived neighborhood characteristics. Medical care costs for all expenses except for pharmacy dispensing were estimated using the standardized relative resource cost algorithm (*9*); pharmacy retail costs were obtained from internal health plan data. Algorithm cost estimate outcomes for healthcare utilization approximate those using Medicare fee schedules, and internal pharmacy cost estimates are similar to retail prices within the local community. Cost estimates represent the total costs to the health system. The Institutional Review Board at Kaiser Permanente Northwest approved the study.

### Study Design and Population

The parent study was designed to estimate all-age, population-based incidence rates of norovirus and other pathogens that contribute to AGE in the United States by using an integrated healthcare delivery system as a surveillance platform (*8*). Using a subset of these data, we conducted a retrospective cohort study of all MAAGE cases identified through the surveillance program along with a comparable group of KPNW medical members who were free from MAAGE for at least 3 months before any non-MAAGE index medical encounter within the health system.

#### Cases

All MAAGE case-patients who sought care during April 1, 2014–September 30, 2016, within the health system were identified using an EHR-based automated extraction program searching for MAAGE-related encounters as identified through codes from the International Classification of Diseases (ICD), 9th Revision (ICD-9) or 10th Revision (ICD-10) and standardized variables from the EHR (*8*). Case-patients could be included more than once if they had separate, distinct episodes of MAAGE separated by >30 days. Episodes of MAAGE represented a continuous illness and could include multiple MAAGE-related encounters. Inclusion and exclusion criteria were minimal at the case-identification stage of the project: patients had to be medical members at the time of the encounter, which served as their index visit; have an ICD-9 or ICD-10 diagnosis related to AGE at an outpatient, urgent care, emergency department, or inpatient encounter; or have a chief complaint of AGE at a telephone encounter (video telehealth visits were uncommon in the health system during this time period). The only patients excluded were those who opted out of research studies during insurance registration (≈0.2% of the KPNW population).

#### Comparators

We selected comparators retrospectively and matched them to MAAGE cases at a 1:1 ratio. As with study cases, comparators had to be current medical members at the time of an index healthcare encounter and were excluded if they had opted out of research. We also required comparators to have no indication of MAAGE in the 3 months before their index encounter. We chose to require comparators to have an index encounter rather than selecting from the general population to minimize the risk of bias from barriers to health services access. It is important to note, however, that the comparison of MAAGE in these analyses is specific to a similar health services–seeking population rather than to the general population. Any non–MAAGE-related visit within the medical system qualified as an index visit. For adults ≥18 years of age at their index encounter, comparators were matched to cases based on gender, age (±2.5 years), and the timing of the index visit (±3 months). We used the same criteria for children but with closer matching on age: children ≤2 years were matched on month of age, and children >2 years were matched on year of age. A single healthcare encounter for each comparator was selected randomly from eligible encounters, which was used as their index encounter, and we matched without replacement.

### Study Outcomes and Control Variables

Study outcomes were all-cause health system costs on the day of and for 30 days after the index encounter, as well as the sum total of costs over these 2 specified times. We also examined separately the total outpatient and pharmacy costs associated with each index encounter and the 30 days after.

We selected control variables that were the most likely observable confounders between MAAGE categorization and health services use: indicators of a history of cancer or diabetes, and diseases of the blood, heart, immune system, kidney, liver, lung, metabolism, or brain. Although we expected lower prevalence among children, we selected the same confounding variables in analyses of both adults and children. We also included self-reported race and ethnicity, as available in the EHR.

### Statistical Analyses

We conducted statistical analyses by using SAS software version 9.4 (SAS Institute, Inc., https://www.sas.com). We conducted separate analyses for adults (≥18 years of age) and children (≤17 years of age). We performed additional analyses, including analyzing data by smaller age subsets (0–4 years, 5–17 years, 18–64 years, and ≥65 years). We compared baseline characteristics between cases and comparators by using χ^2^ tests for categorical variables and *t*-tests for continuous variables.

We modeled outcomes by using general linear models with a gamma distribution and a log link. Cost data are typically nonnormal and highly skewed, and those models can well accommodate these types of data. All comparisons accounted for the matching structure of the data and potential clustering within persons (for cases with >1 MAAGE episode). We calculated and presented adjusted mean differences (AMDs), which are model-based estimates of the mean difference between cases and comparators (MAAGE–comparator), adjusting for the likely confounders included in regression models. We calculated quasi-likelihood modifications to the Akaike Information Criteria (QICs) as model goodness of fit statistics (*10**,**11*).

Our primary analyses included all cases and matched comparators. Because we anticipated outliers in the data, we conducted sensitivity analyses to identify and exclude overly influential outliers (*12*) to determine their influence on our primary analyses. All tests of statistical inference used a 2-sided α = 0.05.

## Results

### Adults

We compiled the baseline characteristics of adult MAAGE cases and matched comparators (Table 1). The mean age of adult cases was 51.6 years (SD +19.4) and the mean age of adult comparators was 50.8 years (SD +19.4). The adult study sample was approximately two thirds female and one third male; 63.7% of case-patients and 63.6% of comparators were female. There were significant differences between adult cases and comparators in race and ethnicity and in almost all underlying conditions, except cancer; case-patients were somewhat more likely than comparators to be Hispanic or non-Hispanic White and having higher rates of all underlying conditions.

**Table 1:** Demographic characteristics and underlying conditions among adult medically attended acute gastroenteritis case-patients and age- and gender-matched comparators in an integrated health system in the Pacific Northwest of the United States, April 1, 2014–September 30, 2016*

EHR-derived measures	Case-patients, n = 31,865†	Comparators, n = 34,265	p value‡
Age, y, mean (+SD)	51.6 (19.4)	50.8 (19.4)	<0.0001
Sex, %			
F	63.7	63.6	NA
M	36.3	36.4	NA
Race/ethnicity, %			
Hispanic	7.1	6.7	<0.001
Non-Hispanic White	82.4	81.3	<0.001
Non-Hispanic other race	8.9	9.9	<0.001
Unknown	1.6	2.1	<0.001
Comorbidity, %§			
Blood disease	4.3	2.9	<0.0001
Cancer	5.0	4.7	0.277
Diabetes	14.4	11.2	<0.0001
Heart disease	18.2	15.0	<0.0001
Immune disease	4.3	3.0	<0.0001
Kidney disease	10.6	8.5	<0.0001
Liver disease	2.1	1.3	<0.0001
Lung disease	13.0	9.7	<0.0001
Metabolic disease	21.7	17.7	<0.0001
Neurologic disease	10.2	7.7	<0.0001

*All participants were Kaiser Permanente Northwest medical members, ≥18 y of age, with ≥1 encounter at the time of the study. EHR, electronic health records; NA, not applicable. †Corresponds to 36,117 episodes; 2,758 MAAGE cases are comparators at some time in the study. ‡Paired t-test or χ^2^ test. §Percentage with diagnosis codes from the International Classification of Diseases, 9th Revision or 10th Revision, in the 12 mo before index encounter date.

Regression analyses of costs among adults (Appendix Table 1) revealed that costs were higher for MAAGE cases than comparators both on the day of their index encounter (AMD = $140; p<0.001) as well as during the 30-day follow-up period (AMD = $296; p<0.001). Accordingly, cases had higher total costs than comparators (AMD = $451; p<0.001). Category-specific costs were higher for cases than comparators in both the outpatient (AMD = $111; p<0.001) and pharmaceutical (AMD = $262; p<0.001) categories.

Differences between adult case-patients and comparators were consistently significantly higher in both the younger (18–64 years) and older (≥65 years) age groups. Comparing case-patients to comparators among older adults (≥65 years), total costs were higher (AMD = $599; p<0.001), both at the index date (AMD = $84; p = 0.004) and during the 30-day follow-up (AMD = $526; p<0.001). We also found that outpatient costs were higher (AMD = $114; p<0.001) as were pharmacy costs (AMD = $280; p<0.001), among cases involving older patients as compared with their comparator counterparts.

Comparing cases to comparators among younger adults, we found that total costs were higher (AMD = $361; p<0.001), both at the index date (AMD = $152; p<0.001) and during the 30-day follow-up (AMD = $196; p<0.001). We also found that outpatient costs were higher (AMD = $106; p<0.001), as were pharmacy costs (AMD = $235; p<0.001), among the younger cases as compared with the comparator patients. Sensitivity analyses identified 2 overly influential outliers in the total cost model and 2 overly influential outliers in the pharmacy cost model. In neither case did removal of those observations substantively change the results; p values remained the same, and the coefficients reduced by 0.8% in the total cost model and 0.3% in the pharmacy cost model.

### Children

We compiled baseline characteristics for child MAAGE cases and matched comparators (Table 2). The mean age of child case-patient s was 6.0 years (SD +5.2) and the mean age of child comparators was 6.3 years (SD +5.3). Forty-seven percent of case-patients and 47.1% of comparators were female; 53% of case-patient s and 52.9% of comparators were male. There were significant differences between child case-patients and comparators in race and ethnicity. Child case-patients were more likely to be Hispanic or non-White than comparators. Child case-patients were also more likely than comparators to have lung disease, although there were no significant differences in other underlying conditions.

**Table 2:** Demographic characteristics and underlying conditions among pediatric medically attended acute gastroenteritis case-patients and age- and gender-matched comparators in an integrated health system in the Pacific Northwest of the United States, April 1, 2014–September 30, 2016*

EHR-derived measures	MAAGE cases, n = 8,558†	Comparators, N = 8,580	p value‡
Mean age, y (+SD)	6.0 (5.2)	6.3 (5.3)	<0.001
Sex, %			
F	47.0	47.1	NA
M	53.0	52.9	NA
Race/ethnicity, %			
Hispanic	18.0	11.7	<0.001
Non-Hispanic White	63.5	70.2	<0.001
Non-Hispanic other race	15.0	14.3	<0.001
Unknown	3.5	3.8	<0.001
Comorbidity, %§			
Blood disease	0.7	0.6	0.384
Cancer	0.2	0.3	0.547
Diabetes	0.4	0.5	0.651
Heart disease	1.4	1.5	0.524
Immune disease	0.4	0.3	0.047
Kidney disease	0.4	0.4	0.800
Liver disease	0.02	0.03	0.655
Lung disease	8.1	6.6	<0.001
Metabolic disease	0.7	0.8	0.528
Neurologic disease	1.8	2.0	0.317

*All participants were Kaiser Permanente Northwest medical members, <18 y of age, with ≥1 encounter at the time of the study. EHR, electronic health records; NA, not applicable †Corresponds to 9,203 episodes; 834 MAAGE cases were comparators at some time in the study ‡Paired *t*-test or χ^2^ test. §Percentage with diagnosis codes from the International Classification of Diseases, 9th Revision or 10th Revision, in the 12 mo before index encounter date.

Regression analyses of costs among children (Appendix Table 2) showed, as for adults, costs were higher for case-patients than for comparators both on the day of the index encounter (AMD = $42; p = 0.001) and during the 30-day follow-up (AMD = $105; p = 0.002), resulting in higher total costs (AMD = $141; p<0.001). Category-specific costs were also higher for case-patients compared with comparators in both the outpatient (AMD = $35; p<0.001) and pharmaceutical (AMD = $40; p = 0.002) categories.

For older children (5–17 years of age), all measured costs were significantly higher for case-patients than comparators. However, for younger children (0–4 years of age), there were no significant differences in total, same-day, or follow-up costs between case-patients and comparators. Among younger children, differences in costs between case-patients and comparators were significantly higher for outpatient costs and pharmacy costs (outpatient AMD = $29; pharmacy AMD = $24; p<0.001 for both). Comparing cases to comparators among older children, total costs were higher (AMD = $358; p<0.001), both at the index date (AMD = $133; p = 0.004) and during the 30-day follow-up (AMD = $214; p<0.001). We also found that outpatient costs were higher (AMD = $47; p<0.001), as were pharmacy costs (AMD = $92; p<0.001), among the older child case-patients compared with their comparator counterparts.

Sensitivity analyses identified 5 overly influential outliers in the total cost model and 4 overly influential outliers in the pharmacy cost model. Removal of those observations did not substantively change the results: p values remained subjectively the same, and coefficients increased by 0.1% in the total cost model and by 11.2% in the pharmacy cost model.

## Discussion

Our data show that medical care expenditures were significantly higher among adult and child healthcare delivery service members identified with MAAGE relative to usual-care medical services among similar healthcare delivery service members, both on the date of an index medical visit and for 30 days following the visit, after controlling for observable differences. This pattern was consistent across total, pharmacy, and outpatient expenditures for all patients ≥5 years of age (total costs did not differ for children 0–4 years of age). Among the cost components contributing to total costs, the magnitude of the difference was largest for pharmacy costs. We also found that follow-up costs were consistently higher than costs accrued on the day of the index encounter. These data fill in gaps in knowledge about the short-term costs of MAAGE among adults and children, particularly in the days following an initial medical encounter. Our findings substantially inform the study of resource burden by incorporating the subsequent, short-term follow-up costs related to a MAAGE episode, which is often omitted in calculating the overall economic burden of MAAGE, thereby underestimating the true burden of MAAGE episodes.

Higher short-term costs associated with MAAGE relative to usual-care medical services may be partly attributable to ongoing pharmacologic intervention, as suggested by differences in pharmacy-related costs. In addition, exacerbation of underlying conditions and illnesses could contribute to the observed differences in cost between those with MAAGE and other patients.

Among adults, incremental total costs associated with MAAGE relative to usual-care medical services were almost twice as high among those ≥65 years of age compared with those 18–64 years of age. When considered as a group, children 0–17 years of age who were identified with MAAGE had higher total costs compared with similar children without MAAGE; however, there were no statistically significant differences in total costs among young children (0–4 years of age). This observation could be because older children may be using medical care less frequently and an episode of AGE would warrant an independent encounter. Conversely, younger children may be using services more frequently and AGE symptoms could be addressed as part of another regular visit.

This study leveraged a large sample of members of an integrated care delivery system and used matched comparators to estimate the associated costs of MAAGE, controlling for likely confounders and measuring MAAGE-related costs in the 30 days following the initial date of presentation. However, among the study’s limitations is that unobserved (and thus uncontrolled) differences between MAAGE case-patients and comparators could have confounded cost estimates, and we observed significant differences between groups after matching. This limitation is inherent to observational studies; however, we controlled for important, observable confounders. Despite matching and the direct control of these observable confounders, biases from unobserved confounders (e.g., income or education levels) likely persist in our estimates. An additional limitation of the study is that findings may not be generalizable beyond the region served by KPNW or to nonintegrated health systems. Although there are similarities across Kaiser Permanente regions, regional differences in member populations and practice patterns limit our ability to generalize to other areas served by Kaiser Permanente. Our findings benefit from the complete capture of health services through the integrated care delivery system, but future research is needed to see if the patterns and magnitude of the economic burden associated with MAAGE vary across healthcare system types, regions, and important patient factors. The data used in our study are 6–8 years old and may not represent current costs. Our analyses adjust to a common year and primarily focus on relative costs between groups, which, although prone to influence from differential changes in costs of medical services over time, are less influenced by general medical-care inflation.

In conclusion, in a large surveillance cohort, we found significantly increased, incremental cost associated with MAAGE-related services relative to usual-care medical services. We noted this increased cost for both adults and for children ≥5 years of age, and those higher costs persisted during the 30-day follow-up period from initial encounters. These findings suggest opportunities to prevent or intervene in the short-term period following a new MAAGE episode to mitigate costs, especially in adults ≥65 years of age.

Appendix More information regarding economic burden of acute gastroenteritis among members of integrated healthcare delivery system, United States, 2014–2016.
