# Supplementary material for: Economic Burden of Acute Gastroenteritis among Members of Integrated Healthcare Delivery System, United States, 2014–2016
Source: Emerg Infect Dis. 2024 May;30(5):968–73. doi: 10.3201/eid3005.230356 (PMC11060443; doi:10.3201/eid3005.230356)
Supplement: Appendix — More information regarding economic burden of acute gastroenteritis among members of integrated healthcare delivery system, United States, 2014–2016. [file 23-0356-Techapp-s1.pdf]

Article DOI: http://doi.org/10.3201/eid3005.230356

EID cannot ensure accessibility for supplementary materials supplied by authors. Readers who have difficulty accessing supplementary content should contact the authors for assistance.

Economic Burden of Acute Gastroenteritis among Members of Integrated Healthcare Delivery System, United States, 2014–2016

Appendix

Appendix Table 1. Short-term mean predicted medical expenditures (2017 USD) of MAAGE episodes among adults (≥18 y) who were Kaiser Permanente Northwest medical members with ≥1 encounter during April 1, 2014–September 30, 2016

	All adults (N* = 73,140)†			Adults 18–64 y (N* = 51,538)‡			Adults ≥65 y (N* = 21,602)§		
**Expenditure category**	MAAGE cases	Comparators	Adjusted diff; p-value¶, QIC	MAAGE cases	Comparators	Adjusted diff; p-value¶, QIC	MAAGE cases	Comparators	Adjusted diff; p-value¶, QIC
**Total cost, mean (95% CI)**	$2,080 (2,017–2,142)	$1,630 (1,581–1,678)	$451 (371–532); <0.001, 147595.7584	$1,716 (1,655–1,777)	$1,356 (1,309–1,403)	$361 (283–439); <0.001, 62546.8588	$2,619 (2,238–3,000)	$2,024 (1,730–2,319)	$599 (417–781); <0.001, 46547.7071
**Timing of total cost**									
On same day	$501 (480–522)	$362 (348–377)	$140 (113–166); <0.001, 65539.7034	$475 (454–496)	$323 (309–337)	$152 (127–179); <0.001, 55385.6281	$502 (372–632)	$419 (309–529)	$84 (23–144); 0.004, 15382.0034
During 30-d follow-up	$1,545 (1,488–1,602)	$1,250 (1,205–1,296)	$296 (222–370); <0.001, 98558.9801	$1,217 (1,162–1,272)	$1,020 (975–1,065)	$196 (125–269); <0.001, 65211.3615	$2,119 (1,769–2,469)	$1,598 (1,334–1,861)	$526 (356–695); <0.001, 47809.3917
**Selected components of total cost**									
Outpatient costs	$506 (500–512)	$396 (391–400)	$111 (104–119); <0.001, 855534.3793	$473 (466–479)	$367 (362–372)	$106 (97–114); <0.001, 230507.5783	$553 (519–588)	$440 (413–468)	$114 (97–130); <0.001, 143198.9770
Pharmacy costs	$652 (633–672)	$395 (383–407)	$262 (237–286); <0.001, 123723.3253	$558 (536–580)	$328 (315–340)	$235 (208–261); <0.001, 52629.2820	$806 (696–917)	$530 (557–604)	$280 (223–339); <0.001, 51097.4559

*N represents number of total MAAGE episodes
†Predicted means calculated at means of the overall adult sample
‡Predicted means calculated at means of the adult sample aged 18–64
§Predicted means calculated at means of the adult sample aged 65+
¶General linear model with gamma distribution and log link; adjusted for all variables in Table 1 (main text).
CI, confidence interval; MAAGE, medically attended acute gastroenteritis; QIC, quality improvement collaborative.

Appendix Table 2. Short-term mean predicted (95% CI) medical expenditures (2017 United States dollars) of MAAGE episodes among children (0–17 y of age) who were Kaiser Permanente Northwest medical members with ≥1 encounter during April 1, 2014–September 30, 2016

	**All children (N* = 18,617)†**			**Children aged 0–4 y (N* = 9,558)‡**			**Children aged 5–17 y (N* = 9,059)§**		
**Expenditure category**	MAAGE cases	Comparators	Adjusted diff; p-value¶, QIC	MAAGE Cases	Comparators	Adjusted diff; p-value¶, QIC	MAAGE Cases	Comparators	Adjusted diff; p-value¶, QIC
**Total cost, mean (95% CI)**	$735 (675–794)	$594 (546–641)	$141 (64–218); <0.001, 18328.6776	$540 (494–585)	$543 (496–591)	−$3 (−70–63); 0.920, 15330.0516	$966 (856–1,076)	$614 (544–683)	$358 (221–493); <0.001, 8353.1631
**Timing of total cost**									
On same day	$282 (263–302)	$240 (223–256)	$42 (17–69); 0.001, 21340.5966	$211 (195–228)	$236 (217–254)	−$24 (−50–1); 0.060, 15941.8937	$372 (333–412)	$241 (216–266)	$133 (84–183); 0.004, 10064.9881
During 30-d follow-up	$449 (396–501)	$344 (304–384)	$105 (38–172); 0.002, 9088.8430	$319 (281–357)	$296 (260–332)	$23 (−30–76); 0.393, 8121.5579	$575 (483–667)	$364 (306–422)	$214 (100–329); <0.001, 4316.5666
**Selected components of total cost**									
Outpatient costs	$307 (300–313)	$272 (266–278)	$35 (26–44); <0.001, 223939.9908	$288 (279–297)	$259 (250–267)	$29 (17–42); <0.001, 99797.9225	$325 (315–335)	$278 (270–287)	$47 (34–60); <0.001, 112966.7246
Pharmacy costs	$100 (78–121)	$61 (48–74)	$40 (13–66); 0.002, 1844.0943	$52 (45–59)	$29 (25–32)	$24 (16–33); <0.001, 4328.0394	$185 (153–217)	$96 (80–112)	$92 (53–131); <0.001, 1260.6387

*N represents number of total MAAGE episodes
†Predicted means calculated at means of the overall sample of children
‡Predicted means calculated at means of the sample of children 0–4 y of age
§Predicted means calculated at means of the sample of children 5–17 y of age
¶General linear model with gamma distribution and log link; adjusted for all variables in Table 2 (main text).
CI, confidence interval; MAAGE, medically attended acute gastroenteritis; QIC, quality improvement collaborative.
